# Bioactive Metabolites from the Deep Subseafloor Fungus *Oidiodendron griseum* UBOCC-A-114129

**DOI:** 10.3390/md15040111

**Published:** 2017-04-07

**Authors:** Marion Navarri, Camille Jégou, Arnaud Bondon, Sandrine Pottier, Stéphane Bach, Blandine Baratte, Sandrine Ruchaud, Georges Barbier, Gaëtan Burgaud, Yannick Fleury

**Affiliations:** 1 Université de Brest, EA 3882, Laboratoire Universitaire de Biodiversité et Ecologie Microbienne, IBSAM, 6 Rue de l’université, 29000 Quimper, France; marion.navarri@univ-brest.fr (M.N.); Camille.jegou@univ-brest.fr (C.J.); Georges.barbier@univ-brest.fr (G.B.); Gaetan.burgaud@univ-brest.fr (G.B.); 2 COrInt, UMR CNRS 6226 & Université de Rennes, 35043 Rennes, France; arnaud.bondon@univ-rennes1.fr (A.B.); sandrine.pottier@univ-rennes1.fr (S.P.); 3 Kinase Inhibitor Specialized Screening facility (KISSf), Station Biologique de Roscoff, Centre National de la Recherche Scientifique (CNRS) USR 3151 (Protein Phosphorylation and Human Diseases), CS 90074, Roscoff CEDEX 29688, France; bach@sb-roscoff.fr (S.B.); baratte@sb-roscoff.fr (B.B.); sruchaud@sb-roscoff.fr (S.R.)

**Keywords:** deep subseafloor fungi, bioactivities, antibacterial, anti-kinase

## Abstract

Four bioactive compounds have been isolated from the fungus *Oidiodendron griseum* UBOCC-A-114129 cultivated from deep subsurface sediment. They were structurally characterized using a combination of LC–MS/MS and NMR analyses as fuscin and its derivatives (dihydrofuscin, dihydrosecofuscin, and secofuscin) and identified as polyketides. Albeit those compounds were already obtained from terrestrial fungi, this is the first report of their production by an *Oidiodendron* species and by the deepest subseafloor isolate ever studied for biological activities. We report a weak antibacterial activity of dihydrosecofuscin and secofuscin mainly directed against Gram-positive bacteria (Minimum Inhibitory Concentration (MIC) equal to Minimum Bactericidal Concentration (MBC), in the range of 100 μg/mL). The activity on various protein kinases was also analyzed and revealed a significant inhibition of CDC2-like kinase-1 (CLK1) by dihysecofuscin.

## 1. Introduction

The ocean harbors a tremendous diversity of habitats, ranging from coastal waters to the deep biosphere, where microorganisms, through their biotic and abiotic interactions, are the major actors of the biogeochemical processes [1,2]. Bacteria, Archaea, and protists are the most commonly documented microorganisms in the marine environment. However, recent studies strongly support the idea that marine microbial communities also host fungi as an important component in different kinds of habitats, including the deep biosphere [3]. Deep subseafloor sediment microbial communities are in constant interactions though the production of a wide array of secondary metabolites, as recently revealed using metatranscriptomics [4,5]. Given that a large number of marine microorganisms can produce a wide array of bioactive metabolites and has consequently received a great amount of attention in the search for natural compounds [6,7,8], the deep biosphere appears to remain an untapped reservoir.

Recently, an exhaustive culture-based approach allowed us to isolate 183 deep subsurface marine fungi [3]. An antimicrobial screening on 110 of those fungal isolates highlighted that 33% of the assayed strains had antimicrobial properties [9]. Among those 110 fungal isolates, 14 were considered as promising based on their antimicrobial activities, and finally the strain *Oidiodendron griseum* CB_36 UBOCC-A-114129 (isolated at 765 m below the sea floor) was selected for further investigation since it seemed to be the bioactive strain with the deepest origin known to date.

In this study, the bioactive metabolites of *O. griseum* CB_36 were investigated using a bioguided fractionation. Four antimicrobial compounds have been characterized using Nuclear Magnetic Resonance (NMR): fuscin (**1**), dihydrofuscin (**2**), dihydrosecofuscin (**3**), and secofuscin (**4**). Surprisingly, although those structures have been known for years from terrestrial fungi [10,11], no biological activity has been ever described to the best of our knowledge for secofuscin and dihydrosecofuscin.

Antimicrobial resistance represents an increasing threat jeopardizing global public health. The antibiotic resistance crisis has been partly attributed to the lack of new drug development by the pharmaceutical industry. The “old” chemical structures discovered in the golden age of antibiotics and abandoned because of their toxicity or lack of efficiency, such as polymyxins, fosfomycin, fusidic acid, or chloramphenicol, are currently being re-investigated for therapeutic use [12]. Therefore, we re-analyzed biological properties of fuscin and its derivatives using recent technologies. We describe herein their antibacterial activity but also their ability to inhibit protein kinase activity. Our results suggest that these “old” known structures may not be obsolete for biotechnological applications and notably for human therapeutics.

## 2. Results

### 2.1. Purification and Characterization of the Bioactive Compounds Produced by Oidiodendron griseum UBOCC-A-114129

**Figure 1:**
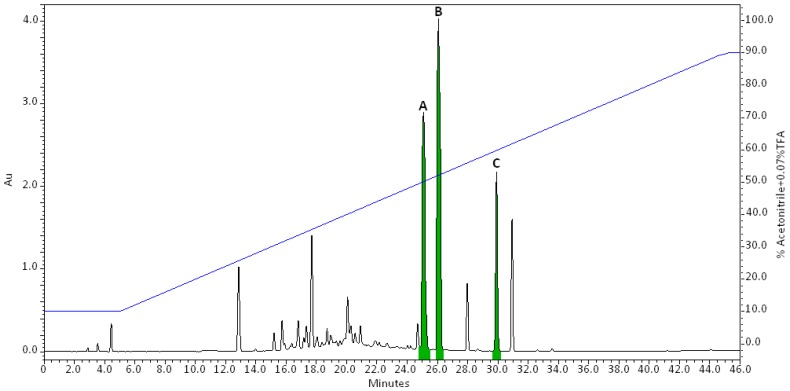
Reverse phase-high-performance liquid chromatography (RP-HPLC) analysis of the F10–90 fractions. Elution was performed using a linear gradient of acetonitrile ACN + 0.07% trifluoroacetic acid (TFA) (blue line). The chromatogram represents the max plot recorded between 220 and 600 nm. Peaks exhibiting antimicrobial activity are overlaid in green. Bioactive compounds were numerated according to their elution order.

Antimicrobial activities were only recovered in the F10–90 extract supporting previous data [9]. The F10–90 extract was afterwards analyzed by Reversed Phase-High-Performance Liquid Chromatography (RP-HPLC) (Figure 1). Fractions were collected along the elution, freeze-dried, resolubilized in 20% Acetonitrile (ACN), and assayed for antimicrobial activity. Only, three fractions eluted from 24 to 32 min exhibited antimicrobial activity against *Enterococcus faecalis* (Figure 1).

Purification of bioactive compounds was performed according to a linear gradient of ACN + 0.07% Trifluoroacetic acid (TFA) and leads to four compounds with antimicrobial properties against *E. faecalis*. LC/ESIMS analyses of Peak A revealed a co-elution of two compounds, herein named **1** (*m*/*z* 277 [M + H]^+^) and **2** (*m*/*z* 279 [M + H]^+^). They were obtained together as a dark orange powder. Compounds **3** (*m*/*z* 279 [M + H]^+^, white powder) and **4** (*m*/*z* 277 [M + H]^+^, dark orange powder) were eluted under Peaks B and C, respectively.

**Figure 2:**
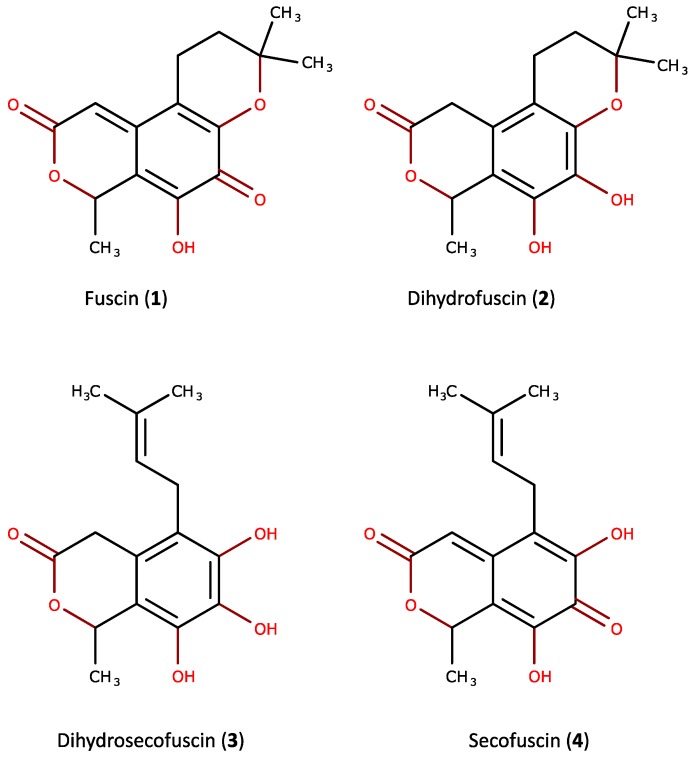
Chemical structures of the bioactive compounds produced by *Oidiodendron griseum* CB_36.

Structural elucidations were conducted via NMR. The bioactive compounds isolated from *O. griseum* UBOCC-A-114129 were identified as fuscin (**1**), dihydrofuscin (**2**), dihydrosecofuscin (**3**), and secofuscin (**4**) (Figure 2). Fuscin and its derivatives characterized here appear as already known fungal compounds. Fuscin and dihydrofuscin have been isolated from *Oidiodendron fuscum* Robak, isolated from soil [10], the phytopathogenic fungus *Potebniamyces gallicola* n. sp. [11] and from *O. griseum*, isolated from soil [13]. Fuscin and dihydrofuscin were described as antibacterial metabolites [10]. 

Interestingly, fuscin and its derivatives were clearly the main amphiphilic metabolites produced by the strain of *O. griseum* since they represented almost 68% of the area of the chromatogram. 

### 2.2. Kinetics of Fuscin Derivative Production

**Figure 3:**
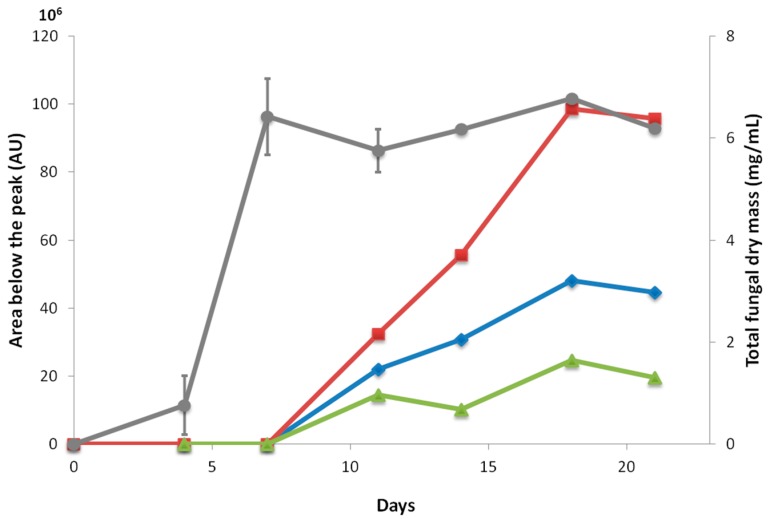
Kinetics of *O. griseum* UBOCC-A-114129 growth (●) and of fuscin and derivatives production resulting from three independent experiments. The latter were quantified by the area below the elution peak detected by RP-HPLC after solid-phase extraction (SPE) extraction of the cell-free supernatant. ■: Dihydrosecofuscin, ♦: fuscin and dihydrofuscin, ▲: secofuscin.

The fungal biomass and the production of fuscin derivatives were quantified over time (Figure 3). Fuscin and its derivatives were only detected when *O. griseum* UBOCC-A-114129 reached the stationary phase of fungal growth. Fuscin and its derivatives were detected after 7 days of culture at 25 °C and their production increased and achieved a maximal production after 18 days of culture. To date, the ecological role of these compounds is still unknown. Nevertheless, such a constitutive production during the stationary phase is commonly described for antimicrobial metabolites, such as citrinin produced by *Penicillium chrysogenum* [14].

### 2.3. Biological Activities

#### 2.3.1. Antimicrobial Activity

We investigated the antimicrobial activity of secofuscin and dihydrosecofuscin since these metabolites (HPLC fractions) exhibited antimicrobial activity against *E. faecalis.* Their minimal inhibitory concentrations (MICs) were defined in accordance with the international standards by Clinical and Laboratory Standards Institute (CLSI guidelines M7-A09) against a panel of nine bacterial targets. A weak antibacterial activity was detected (MIC in the range of 100 μg/mL). To attain insight into the antibacterial mode of action (bactericidal versus bacteriostatic), 5 μL of suspension at MIC and MIC/2 was dropped onto tryptone soy agar (TSA) medium and incubated at the optimal temperature for bacterial target growth. These compounds were not only bacteriostatic but also bactericidal at MICs for *Staphylococcus aureus*, methicillin-resistant *S. aureus* (MRSA), *Acinetobacter baumannii*, and *Klebsiella pneumoniae* (Table 1).

#### 2.3.2. Disease-Related Kinase Inhibition

Dihydrosecofuscin obtained from *O. griseum* UBOCC-A-114129 was assayed on a panel of 11 disease-related kinases. It was first assayed at a final concentration of 50 μg/mL. When enzymatic inhibition was lower than 50% (half maximal inhibitory concentration (IC_50_) > 50 μg/mL), dihydrosecofuscin was considered inactive (Table 2). At 50 μg/mL, dihydrosecofuscin was able to inhibit the activity of the CDC2-like kinase-1 (CLK1) by 83%.

**Figure 4:**
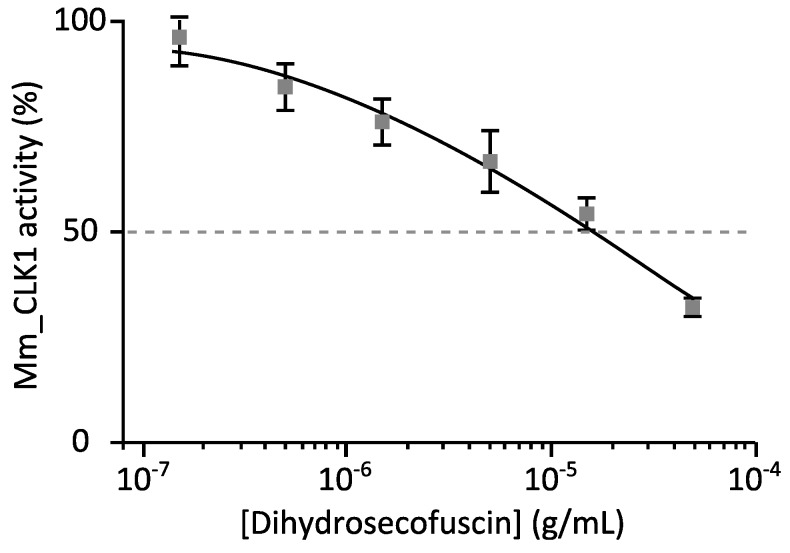
Effect of diyhdrosecofuscin on the catalytic activity of Mm_CLK1. Recombinant GST-CLK1 was assayed in the presence of increasing concentrations of diyhdrosecofuscin. Kinase activities are expressed in % of maximal activity, i.e., measured in the absence of inhibitor (mean ± standard deviation (SD), *n* = 3).

When kinase inhibition was higher than 50%, the IC_50_ was defined from the dose–response curves using a wide range of concentrations (usually from 0.015 μg/mL to 50 μg/mL) (Figure 4). The IC_50_ value of diyhdrosecofuscin against CLK1 was estimated at 15.6 μg/mL.

## 3. Discussion

Ocean covers almost 70% of the earth surface with a wide array of contrasting habitats harboring complex microbial communities. Marine microorganisms were proven to be a great reservoir of secondary bioactive metabolites [15]. Among them, marine fungi represent a potential new reservoir of bioactive natural and sustainable products [16,17]. Deep-sea and subseafloor fungi are the least studied marine fungi and therefore may appear as an untapped diversity with biotechnological potential [18,19]. Indeed, deep sea fungal communities can feed natural compound libraries with putatively novel structures and mode of actions [20].

To reveal the chemical structure of the bioactive metabolites, we investigated the deepest fungal strain (765 m below the sea floor), *O. griseum* UBOCC-A-114129. Fuscin (**1**), dihydrofuscin (**2**), dihydrosecofuscin (**3**), and secofuscin (**4**) were structurally characterized from *O. griseum* UBOCC-A-114129. These compounds have already been described from terrestrial *O. fuscum* [10], *O. griseum* [13], and *P. gallicola* n. sp. [11]. However, to the best of our knowledge, this is the first report of (i) the production of fuscin and its derivatives by a marine fungus in the meantime and (ii) the antimicrobial activity of secofuscin and dihydrosecofuscin.

The production of fuscin (**1**), dihydrofuscin (**2**), dihydrosecofuscin (**3**), and secofuscin (**4**) occurred after the fungus has completed his growth and can thus be qualified as secondary metabolites [21]. Secondary metabolites are small molecular weight molecules produced by microorganisms to compete with environmental stressing conditions such as temperature, pressure, salinity, and those arising from other microorganisms [22]. Some of them could be beneficial as antimicrobial or antitumor activities, whereas other could be deleterious to humankind (e.g., mycotoxins) [23]. Dihydrosecofuscin has been previously described as a biosynthetic precursor of fuscin [24]. It was here the most produced metabolite and appeared to co-occur with the other derivatives. This is the first report on their co-production in an *Oidiodendron* species. Although fuscin and its derivatives appear as the major metabolites produced during the stationary phase, their ecological role remains to be clarified. The chemical structure of those compounds suggested a polyketide, a terpenoid structure, or a hybrid of both, as suggested by Birch and his colleagues [25]. Previous investigation of genes coding polyketides synthases and terpene synthases revealed the unique presence of polyketide synthases in the *O. griseum* UBOCC-A-114129 genome [3] and thus likely highlighted the polyketide nature of the bioactive metabolites produced by this isolate.

Although fuscin (**1**) and dihydrofuscin (**2**) were previously investigated for antimicrobial activities, no data were available regarding dihydrosecofuscin (**3**) and secofuscin (**4**). MIC values reflected a weak but significant antimicrobial activity. A bactericidal mode of action was also revealed against various Gram-positive bacterial targets.

Finally, dihydrosecofuscin showed in vitro inhibition of kinase activity against CLK1. *Penicillium terrestre*, a marine fungus isolated from marine sediment, has also been described for its anti-kinase activity. A 35% inhibition of tyrosine kinases was observed when incubated with 3 μg/mL of the bioactive metabolite terrestrol G synthetized by *Penicillium terrestre* [26]. Therefore, dihydrosecofuscin weakly inhibited CLK1 activity with an IC_50_ of 15.6 μg/mL. Since this kinase has been reported to be a relevant target for Alzheimer’s disease treatment, dihydrosecofuscin may provide a structural pattern for developing new therapeutic drugs.

## 4. Materials and Methods

### 4.1. Fungal Isolation and Identification

Isolation and identification of the fungal isolate selected in this study has been described in an earlier report [3]. Briefly, sediment samples were plated onto five low nutrient media with or without sea salts, at different temperatures and at different hydrostatic pressures. Subcultures of each colony were performed to obtain pure cultures. Purified isolates were then cryo-preserved on beads at −80 °C. 

The sequence analysis of 18S rDNA and internal transcribed spacer (ITS) genetic markers revealed *O. griseum* as the nearest relative of our strain with 99% of similarity in GenBank database. The 18S and ITS rDNA sequence were respectively deposited in Genbank under the accession number KM222226 and KM232506, respectively. The *O. griseum* isolate studied here has been deposited in the Université de Bretagne Occidentale Culture Collection under the UBOCC-A-114129 number (www.univ-brest.fr/ubocc).

### 4.2. Cultivation Method and Fermentation

One cryobead containing a spore suspension of the preserved fungus was plated onto potato dextrose agar (PDA) and incubated at 25 °C for 10 days for revitalization. Then, the strain was subcultured onto PDA for 14 days at 25 °C. Six 250 mL Erlenmeyer flasks, containing 50 mL of Potato Dextrose Broth PDB complemented with 0.2% agar (PDA 0.2%), were inoculated with 2 plugs with a 4-mm diameter cut from the edge of the actively growing culture that was 14 days old. They were incubated under rotary shaking (100 rpm) at 25 °C for 21 days.

### 4.3. Kinetics of Biomass and Metabolite Production

The production of biomass and bioactive metabolites were assessed for 21 days. At 4, 7, 11, 14, 18, and 21 days of incubation, antimicrobial assays were performed using the well diffusion method [9] before extraction of the culture. The culture was filtered on Whatman filter paper (No. 1) (Fisher, Illkirch, France) under vacuum to separate biomass from the culture medium. The biomass was dried in a 58 °C incubator for at least one week and was weighed to draw the kinetic curve. The culture medium was centrifuged (8000× *g*, 20 min, 4 °C) to pellet residual biomass. The supernatant (25 mL) was loaded and fractionated onto a Solid Phase Extraction C-18 column. The first elution step was performed using 10% acetonitrile +0.07% TFA) (Fractions 0–10). The second elution step was conducted using 90% acetonitrile +0.07% TFA and led to Fractions 10–90. Fractions 0–10 and 10–90 were freeze-dried and suspended into 2.5 mL of 20% acetonitrile to obtain extracts 10 times, concentrated at, respectively, 1 mg/mL and 4 mg/mL (±10%)

### 4.4. Bio-Guided Isolation 

Antimicrobial assays were performed on *E. faecalis* CIP-A-186 as the most sensitive target of this fungal strain [9]. An overnight culture of the target was performed in tryptone soy broth. This culture was then diluted and included in TSA to obtain 1.0 × 10^6^ cfu/mL. Finally, 20 μL crude extracts or fractions were dropped into wells, previously performed in the TSA with *E. faecalis*. Inhibition diameter was measured after 24 h of incubation at 37 °C.

### 4.5. Purification of Bioactive Compounds

Cell-free culture supernatant resulting (25 mL) from a 14-day-long incubation culture of *O. griseum* UBOCC-A-114129 was used and subjected to the extraction procedure previously described (i.e., C-18-SPE). A 10× concentrate of Fractions 10–90 was fractionated (each 2 min) on a semi-preparative column C-18-reverse-phase HPLC (Uptisphere strategy; 5 μm; 250 × 10 mm; 4 mL/min, Interchim, Montluçon, France). A gradient from 20% to 90% acetonitrile +0.07% TFA in water +0.1% TFA over 35 min was used and allowed the isolation of 19 fractions. Antimicrobial activity of those fractions was assessed. The antimicrobial compounds in the F10-90 were then purified using a gradient from 36% to 45% acetonitrile +0.07% TFA in water +0.1% TFA over 18 min (data not shown). It yielded to the isolation of Compounds **1** and **2,** which were co-eluted in Fractions 1 (13.8 min and 1.2 mg), **3** (15.3 min and 2.5 mg), and **4** (23.1 min and 0.8 mg) with antimicrobial activities.

### 4.6. Characterization/Spectral Data

The pure compounds were further characterized using liquid chromatography–mass spectrometer (LC–MS) and NMR. The LC–MS analyses were conducted using an ultra Performance Liquid Chromatography (UPLC) system (Acquity H Class Bio, Waters, Milford, MA, USA) coupled with detection by spectrophotometry (PDA eλ detector, Waters) and mass spectrometry (QuattroMicro). Elutions were performed on a C-18 column (BEH, 2.1 × 50 mm, 1.7 μm) using a linear gradient of acetonitrile (0.1% Formic Acid). All the LC–MS instruments and column come from Waters Corporation.

^1^H NMR measurements were carried out on a Bruker AVANCE 500 spectrometer (Bruker, Wissembourg, France) with a TCI cryoprobe. The spectra were recorded at 298 K. Homo-nuclear (Correlation Spectroscopy COSY), Total Correlation Spectroscopy (TOCSY), and hetero-nuclear (Heteronuclear Single Quantum Coherence (HSQC), Heteronuclear Multiple Bond Correlation (HMBC), and HSQC–TOCSY) standard pulse sequences of the Bruker database were used. Samples were solubilized in DMSO-*d*_6_ and chemical shifts were expressed as ppm.

### 4.7. Biological Activities

#### 4.7.1. Minimal Inhibitory Concentrations (MICs)

MICs of Fraction 1, dihydrosecofuscin (**3**) and secofuscin (**4**), and commercial fuscin (Adipogen Life Science, San Diego, CA, USA), were determined using micro-broth dilution methods on *Enterococcus faecalis* ATCC 29212, vancomycin-resistant *Enterococcus faecium* BM4147 (VRE), *Staphylococcus aureus* ATCC 29213, methicillin-resistant *S. aureus* (MRSA), and *Streptococcus equinus* NRRL-B-4268 for Gram-positive bacteria, *Acinetobacter baumannii* CIP70.34T, *Escherichia coli* ATCC 25922, *Klebsiella pneumoniae* ATCC8045, and *Pseudomonas aeruginosa* ATCC 27853 representing Gram-negative bacteria. Experiments were performed as described in CLSI standard M07-A9: methods for dilution antimicrobial susceptibility tests for bacteria that grow aerobically [27]. Briefly, a 96-well microplate containing a 2-fold serial dilution of pure compounds (50 μL) ranging from 512 μg·mL^−1^ to 1 μg·mL^−1^ was prepared. Targets were cryo-preserved at −20 °C. Strains were streaked onto TSA and incubated for 24 h at 37 °C. One colony was suspended into 5 mL of Mueller–Hinton agar and incubated 4 h at 37 °C under rotary shaking. A bacterial suspension (1.0 × 10^6^ cfu/mL) was performed in Mueller–Hinton 2× and dispensed (50 μL) in the previous microplates. Finally, the bacterial concentration reached 5.0 × 10^6^ cfu/mL, and antibiotic final concentrations ranged from 256 μg/mL to 0.5 μg/mL. To validate our results, we verified that the MICs of the positive controls was in agreement with MICs listed by CLSI [27].

#### 4.7.2. Kinase Assays

Kinase activities were assayed in an appropriate kinase buffer, with either protein or peptide as a substrate in the presence of 15 μM [γ-^33^P] ATP (3000 Ci/mmol; 10 mCi/mL) in a final volume of 30 μL following the assay described in [28]. Controls were performed with appropriate dilutions of dimethylsulfoxide. Full-length kinases are used unless specified. Peptide substrates were obtained from Proteogenix (Oberhausbergen, France). The inhibitory activity of dihydrosecofuscin (**3**) was assayed on 11 disease-related kinases incubated in an appropriate buffer: (i) DYRK1A (dual-specificity tyrosine phosphorylation-regulated kinase-1A) from *Rattus norvegicus*; (ii) murine CLK1 (CDC2-like kinase 1); (iii) human CDK9/CyclinT (cyclin-dependent kinase 9); (iv) human CDK5/p25; (v) human CDK2/CyclinA; (vi) GSK-3 (glycogen synthase kinase-3α/β) purified from porcine brain; (vii) CK1 (Casein Kinase 1) purified from porcine brain, (viii) the orthologue of CK1 from *Leishmania major*; (ix) human PIM1; (x) human haspin; and (xi) human RIPK3 (receptor-interacting protein kinase-3) (see Supplementary Materials for details on the kinase assays).

## 5. Conclusions

In this study, we characterized four bioactive compounds produced by *O. griseum*, isolated from a sample collected at 765 m below the sea floor. To our knowledge, this strain is the deepest subseafloor isolate ever studied for biological activities. Although all compounds had been previously described from terrestrial fungus, two of them, dihydrosecofuscin and secofuscin, had not been previously described as bioactive. Here we investigated their biological activities and showed their antibacterial activities against Gram-positive bacteria, with a bactericidal mode of action. Moreover, dihydrosecofuscin inhibited CLK1 kinase activity with an IC_50_ of 15.6 μg/mL, highlighting a possible interest for putative applications in human disease treatment such as Alzheimer’s. Such compounds, especially dihydrosecofuscin, could represent new structural patterns in the search for new bioactive compounds to fight antimicrobial resistance and neurodegenerative disease threats. Although no new structures were revealed here for *O. griseum* UBOCC-A-114129, the collection of deep subsurface isolates still represents an untapped reservoir of bioactive compounds since many other promising isolates remain to be screened for their secondary metabolites.

## Figures and Tables

**Table 1:** Antimicrobial activity of isolated compounds. Minimal inhibitory concentrations (MICs) were determined according the clinical and laboratory standards Institute (CLSI) guidelines M7-A09. Controls for Gram-negative and Gram-positive bacteria were polymixin B and erythromycin, respectively. NA: Not Assayed. (*) indicates that a bactericidal mode of action was highlighted.

		MIC (μg/mL)	
	Dihydrosecofuscin	Secofuscin	Control
*Enterococcus faecalis* ATCC 29212	128–256	>256	4
Vancomycin Resistant *Enterococcus faecium* BM4147	128–256	>256	NA
*Staphylococcus aureus* ATCC 29213	128 *	256 *	1–2
Methicillin-resistant *Staphylococcus aureus*	128 *	≥256	NA
*Streptococcus equinus* NRRL-B-4268	64–128	256	NA
*Cinetobacter baumannii* CIP70.34T	128–256 *	≥256	NA
*Escherichia coli* ATCC 25922	>256	>256	4
*Klebsiella pneumoniae* ATCC 8045	128–256 *	>256	NA
*Pseudomonas aeruginosa* ATCC 27853	≥256	≥256	4

**Table 2:** Residual kinase activity (%) in the presence of dihydrosecofuscin (50 μg/mL).

Protein Kinases	Dihydrosecofuscin
Rn_DYRK1A	63
Mm_CLK1	**17**
Hs_CDK9/CyclinT	69
Hs_CDK5/p25	57
Hs_CDK2/CyclinA	55
Ssc_GSK3a/b	57
Ssc_CK1	72
Lm_CK1	89
Hs_PIM1	86
Hs_Haspin	60
Hs_RIPK3	67

Bold values showed inhibition of kinase activity at 50 μg/mL.

## Supplementary Materials

The following are available online at www.mdpi.com/1660-3397/15/4/111/s1: NMR spectral data of identified compounds and Buffer composition for anti-kinase activity.
