# Supplementary material for: Bioactive Metabolites from the Deep Subseafloor Fungus Oidiodendron griseum UBOCC-A-114129
Source: Mar Drugs. 2017 Apr 7;15(4):111. doi: 10.3390/md15040111 (PMC5408257; doi:10.3390/md15040111)
Supplement: Supplementary file 1 [file marinedrugs-15-00111-s001.pdf]

Supplementary Data

1. *Nuclear magnetic resonance (NMR) spectral data*

	Fuscin		Dihydrofuscin	
Atome	δ [13]C	δ [1]H	δ [13]C	δ [1]H
2	76,9		75,6	
3	31,5	1,87 (br t) J=6,7Hz	32,6	1,85 (br t) J=6,4Hz
4	18	2,61 (m)	18,7	2,51 (m) et 2,61 (m)
5	116,4		109,9	
6	147,1		141,1	
7	175,5		130,5	
8	142,1		137,3	
9	114,4		114,1	
10	72,5	5,73 (q) J=6,7Hz	74,3	5,83 (q) J=6,8Hz
12	163,0		170,9	
13	117,2	6,35 (s)	30,6	3,43 (d) J=19,8Hzet 3,66 (d) J=19,8Hz
14	139,9		118,0	
15	26,2	1,39 (s)	26,3	1,33 (s)
16	26,6	1,42 (s)	27,0	1,37 (s)
17				5,32 (s)
18		6,90 (br s)		5,29 (s)
19	22,4	1,60 (d) J=6,7Hz	22	1,57 (d) J=6,8Hz

	Secofuscin		Dihydrosecofuscin	
Atome	δ [13]C	δ [1]H	δ [13]C	δ [1]H
2	72,7	5,76 (q) J=6,6Hz	74,0	5,84 (q) J=6,8Hz
3	117,1		115,1	
4	141,2		138,3	
5	175,3		130,5	
6	145,8		142,1	
7	121,3		115,8	
8	139,4		118,7	
9	121,2	6,52 (s)	31,3	3,49 (d) J=19,6Hz et 3,76 (d) J=19,6Hz
10	162,6		170,6	
11	22,6	1,60 (d) J=6,6Hz	21,9	1,57 (d) J=6,8Hz
12		6,62 (s)		5,24 *(br s)
13				5,32* (br s)
14		6,67 (s)		5,46* (br s)
15	23,5	3,35 (br d) J=6,8Hz	25,0	3,27 (br d) J=7,0Hz
16	120,2	5,07 (tspt) J=6,8Hz et J=1,5Hz	120,9	5,14 (tspt) J=7,0Hz et J=1,5Hz
17	134,3		136,6	
18	18,1	1,78 (br d) J=1,5Hz	18,1	1,84 (br d) J=1,5Hz
20	25,7	1,71 (br d) J=1,5Hz	25,8	1,78 (br d) J=1,5Hz
			*may be interchanged	

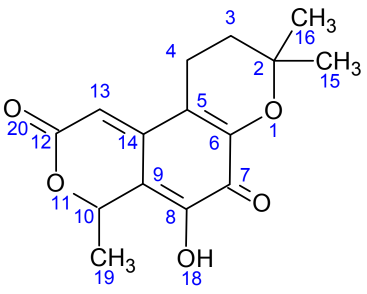

Fuscin

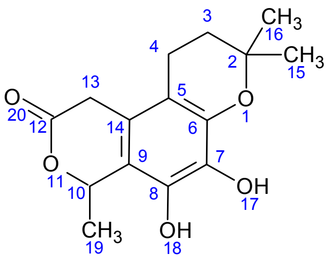

Dihydrofuscin

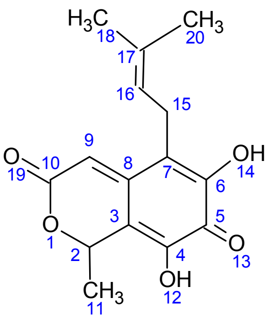

Secofuscin

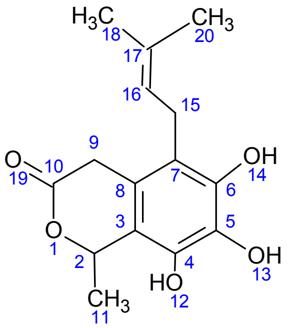

Dihydrosecofuscin

2. *Kinase assays*

The following buffers were used in this study: **Buffer A:** 10 mM $MgCl_2$, 1 mM EGTA, 1 mM DTT, 25 mM Tris-HCl pH 7.5, 50 µg/mL heparin; **Buffer B:** 60mM β-glycerophosphate, 30 mM p-nitrophenyl-phosphate, 25 mM MOPS (pH 7), 5 mM EGTA, 15 mM $MgCl_2$, 1mM DTT, 0.1 mM sodium orthovanadate; **Buffer H:** MOPS 25 mM pH 7.5, 10 mM $MgCl_2$; **Buffer R:** 1.67 mM MOPS pH 7.2, 0.83 mM β-glycerophosphate, 1.33 mM $MgCl_2$, 0.83 mM $MnCl_2$, 0.33 mM EGTA, 0.13 mM EDTA, 16.67 µg/mL BSA, 0.017 mM DTT.

The kinases were assayed using the following experimental conditions: **RIPK3** (human, recombinant, expressed by baculovirus in Sf9 insect cells) was assayed in buffer R with 0.1 µg/µL of MBP as substrate; ***Hs*****PIM1**_(human proto-oncogene, recombinant, expressed in bacteria) was assayed in buffer B with 0.8 µg/µL of histone H1 (Sigma #H5505) as substrate; ***Hs*****Haspin-kd** (human, kinase domain, amino acids 470 to 798, recombinant, expressed in bacteria) was assayed in buffer H with 0.007 µg/µL of Histone H3 (1-21) peptide (ARTKQTARKSTGGKAPRKQLA) as substrate; ***Hs*****CDK2/CyclinA**_(cyclin-dependent kinase-2, human, kindly provided by Dr. A. Echalier-Glazer, Leicester, UK) was assayed in buffer A (+ 0.15 mg/mL of BSA + 0.23 mg/mL of DTT) with 0.8 µg/µL of histone H1 as substrate; ***Hs*****CDK9/CyclinT** (human, recombinant, expressed by baculovirus in Sf9 insect cells) was assayed in buffer A (+ 0.15 mg/mL of BSA + 0.23 mg/mL of DTT) with 0.27 µg/µL of the following peptide: YSPTSPSYSPTSPSYSPTSPSKKKK, as substrate; ***Hs*****CDK5/p25** (human, recombinant, expressed in bacteria) was assayed in buffer B, with 0.8 µg/µL of histone H1 as substrate; ***Ssc*****GSK-3α/β** (glycogen synthase kinase-3, porcine brain, native, affinity purified) was assayed in buffer A (+ 0.15 mg/mL of BSA + 0.23 mg/mL of DTT), with 0.010 µg/µL of GS-1 peptide, a GSK-3-selective substrate (YRRAAVPPSPSLSRHSSPHQSpEDEEE, "Sp" stands for phosphorylated serine); ***Ssc*****CK1δ/ε** (casein kinase 1δ/ε, porcine brain, native, affinity purified) was assayed in buffer B, with 0.022 µg/µL of the following peptide: RRKHAAIGSpAYSITA as CK1-specific substrate; ***Rn*****DYRK1A-kd** (*Rattus norvegicus*, amino acids 1 to 499 including the kinase domain, recombinant, expressed in bacteria, DNA vector kindly provided by Dr. W. Becker, Aachen, Germany) was assayed in buffer A (+0.5 mg/mL of BSA + 0.23 mg/mL of DTT) with 0.033 µg/µL of the following peptide: KKISGRLSPIMTEQ as substrate; ***Mm*****CLK1** (from *Mus musculus*, recombinant, expressed in bacteria) was assayed in buffer A (+ 0.15 mg/mL of BSA + 0.23 mg/mL of DTT) with 0.027 µg/µL of the following peptide: GRSRSRSRSRSR as substrate; ***Lm*****CK1** (from *Leishmania major*, recombinant, expressed in bacteria) was assayed in buffer B (adjusted at pH 8) with 0.028 µg/µL of the following peptide: RRKHAAIGSpAYSITA as CK1-specific substrate.
